# Progesterone-Mediated Enhancement of Hepatitis E Virus Replication in Human Liver Cells

**DOI:** 10.1128/mBio.01434-21

**Published:** 2021-06-22

**Authors:** Harini Sooryanarain, S. Ansar Ahmed, Xiang-Jin Meng

**Affiliations:** a Center for Emerging, Zoonotic and Arthropod-borne Pathogens, Virginia Polytechnic Institute and State University, Blacksburg, Virginia, USA; b Department of Biomedical Sciences and Pathobiology, Virginia-Maryland College of Veterinary Medicine, Virginia Polytechnic Institute and State University, Blacksburg, Virginia, USA; Indiana University Bloomington

**Keywords:** PGRMC1/2 receptor, pregnancy, progesterone, type III interferon, virus replication, hepatitis E virus

## Abstract

Progesterone is crucial for the maintenance of pregnancy. During pregnancy hepatitis E virus (HEV) infection is associated with increased fulminant hepatic failure and mortality rates. In this study, we determined whether progesterone modulates HEV replication and HEV-induced innate cytokine response in Huh7-S10-3 human liver cells. We first demonstrated that Huh7-S10-3 liver cells expressed SH3-domain-containing progesterone receptor membrane component (PGRMC)1/2 receptors involved in the progesterone nonclassical signaling pathway, while the classical progesterone receptor isoforms progesterone receptor-A and -B protein levels were undetectable. We showed that the genotype 3 HEV (strain P6) induced mRNA expression of type III interferon (IFN-λ1), but not other innate cytokines in Huh7-S10-3 cells. Pretreatment with progesterone at concentrations of 80 nM, 160 nM, or 480 nM, which are the physiological concentrations typically seen in the first- to third-trimester during pregnancy, significantly increased HEV replication in Huh7-S10-3 cells. However, pretreatment of cells with progesterone (80 nM) did not affect the level of HEV-induced IFN-λ1 mRNA expression. We further showed that loss of PGRMC1/2 receptors by small interfering RNA (siRNA) knockdown leads to an increase in HEV-induced IFN-λ1 expression levels at early time points via the extracellular signal-regulated kinase pathway and thus resulted in a reduced level of HEV replication. Collectively, the results indicated that progesterone-mediated modulation of HEV replication in human liver cells is plausibly through SH3-domain containing proteins such as PGRMC1/2, but not likely through immunomodulation of HEV-induced interferon response in liver cells. The results have important implications in understanding the underlying mechanisms of high mortality and fulminant hepatitis in HEV-infected pregnant women.

## INTRODUCTION

Hepatitis E virus (HEV) is a nonenveloped positive-sense RNA virus (1). HEV is classified in the family *Hepeviridae*, which consists of two distinct genera, the genus *Orthohepevirus*, comprising four species (*Orthohepevirus A*, *B*, *C*, and *D*), and the genus *Piscihepevirus*, containing a single virus from fish (the cutthroat trout virus). The species *Orthohepevirus A* currently has at least eight genotypes (2), of which HEV genotypes 1 to 4 are of significant importance to human health (3). Molecular characterization of HEV genotypes 1 to 4 strains have revealed a substantial genetic diversity worldwide (4).

HEV infection in humans usually results in a self-limiting acute viral hepatitis (5). However, the mortality rate increases to 20–30% during pregnancy in women predominantly associated with genotype 1 HEV infection (3, 5, 6). Adverse pregnancy outcomes, including miscarriage and stillbirths, have also been reported in pregnant rabbits experimentally infected with genotype 3 and 4 HEVs (7–9). HEV-infected pregnant women also develop a higher rate of acute fulminant hepatic failure (6, 10). Higher levels of HEV RNA and seropositivity were observed in pregnant women with fulminant hepatic failure than in nonpregnant women with fulminant hepatic failure (11). Studies from India showed that HEV-infected pregnant women with fulminant hepatic failure had an increased maternal mortality rate (11, 12) and worse fetal outcomes than HEV-negative pregnant women (11).

Progesterone plays an important role in the maintenance of pregnancy (13). The physiological concentration of progesterone in serum varies (∼0.6 to 900 nM) depending on gender, and in females the phase of the menstrual cycle and stage of pregnancy (Table 1). Progesterone binds to the progesterone receptor (PR-A and PR-B) to mediate the classical signaling pathway (14) and uses the progesterone receptor membrane component (PGRMC) to mediate the nonclassical signaling pathway (15). A recent study revealed that the frequency of progesterone receptor mutation (PROGINS) was 30% in HEV-seropositive patients compared to only 14% in HEV-seronegative patients (16). Additionally, both maternal and fetal mortality in HEV-positive fulminant hepatic failure patients increased in PROGINS carriers (17).

**TABLE 1:** Physiological levels of progesterone in serum according to gender, menstrual cycle phase, and pregnancy status

Gender or stage	Range (ng/ml)^*a*^	Concn used in the study
Males	≤0.2	0.25 ng/ml (0.8 nM PRO)
Postmenopausal women	≤0.2	
Follicular phase	≤0.89	
Ovulation phase	≤12	2.5 ng/ml (8 nM PRO)
Luteal phase	1.8–24	
First trimester	11–44	25 ng/ml (80 nM PRO)
Second trimester	25–83	50 ng/ml (160 nM PRO)
Third trimester	65–290	150 ng/ml (480 nM PRO)

a Clinical progesterone level in serum according to the Mayo Clinic (https://www.mayocliniclabs.com/test-catalog/Clinical+and+Interpretive/8141) and the University of Rochester (https://www.urmc.rochester.edu/encyclopedia/content.aspx?ContentTypeID=167&ContentID=progesterone).

HEV is known to escape the host innate response (18). Various host cellular factors have been shown to act both as proviral and restrictive factors during HEV infection (19). Studies have also shown that HEV induces higher levels of type III interferon (IFN) both in *in vitro* cell culture systems (20) and in sera of patients with acute HEV infection (21). Studies have shown that HEV-infected pregnant women have an impaired immune response (22) and that increased levels of progesterone were detected in HEV-positive pregnant women with fulminant hepatic failure (11). However, the underlying mechanism behind the role of progesterone in the manifestation of severe hepatitis during pregnancy remains unknown. Therefore, in this study we examined whether the progesterone-mediated signaling pathway influences HEV replication by modulating the HEV-induced innate response in human liver cells.

## RESULTS

### Huh7-S10-3 liver cells express nonclassical progesterone receptor PGRMC1/2 but lack classical progesterone receptor PR-A/B.

**FIG 1:**
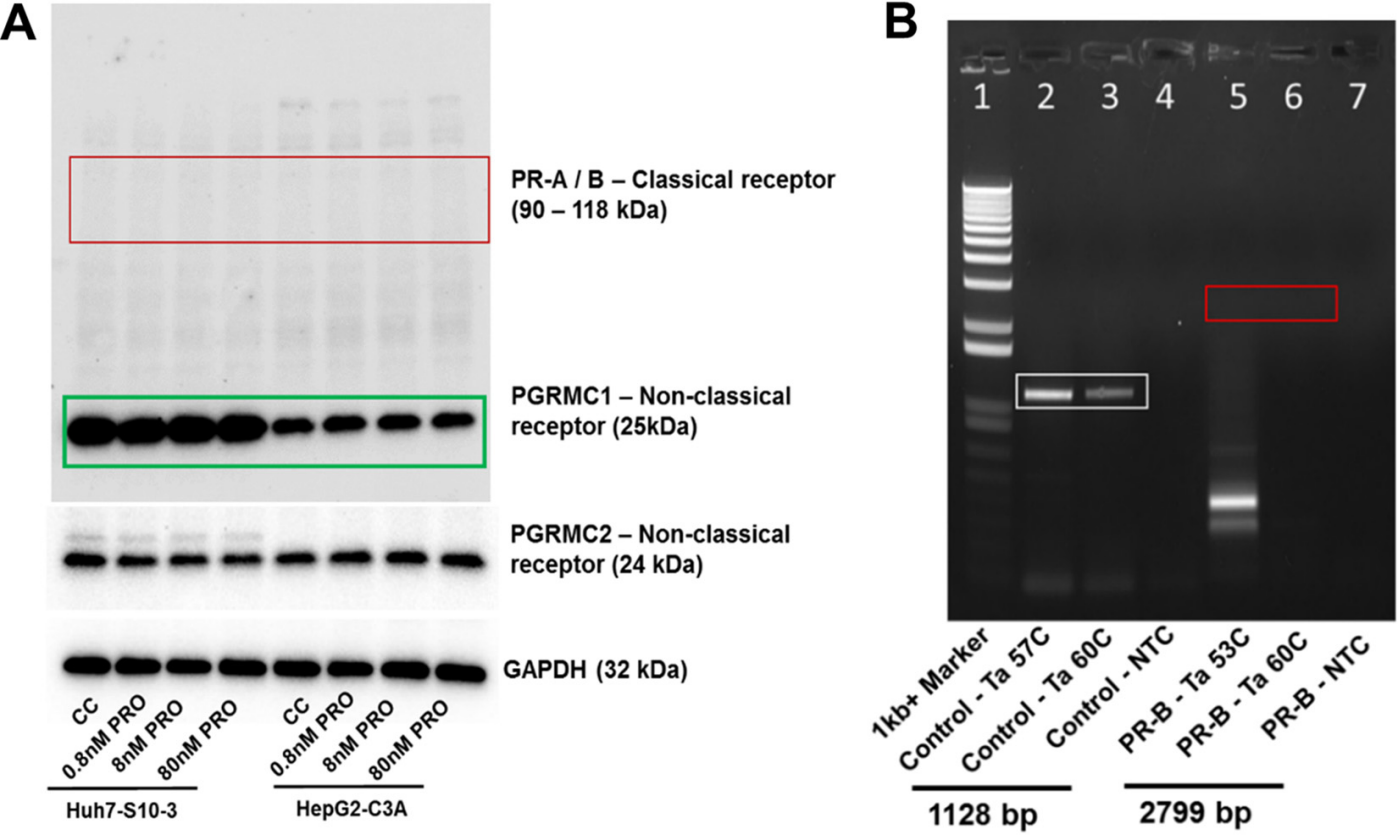
Expression profile of progesterone receptor in human liver cells. (A) Western blot analysis of progesterone receptors, both classical receptor (PR-A, and PR-B) and nonclassical receptor (PGRMC1/2), in Huh7-S-10-3 liver cells and HepG2-C3A liver cells in the presence and absence of various concentrations of progesterone. (B) RT-PCR detection of progesterone receptor PR-B CDS, as well as the control G protein-coupled estrogen receptor (GPER) CDS in Huh7-S10-3 liver cells.

The expression profile of progesterone receptors in human hepatocytes is unclear. The results from Western blot analyses showed that both Huh7-S10-3 and HepG2-C3A cells expressed nonclassical progesterone receptor, progesterone receptor membrane component (PGRMC)-1 and -2 proteins, while the classical progesterone receptor isoforms progesterone receptor (PR)-A and -B protein levels were undetectable (Fig. 1A).

To further confirm that the absence of PR-A, and PR-B protein by Western blotting is not due to low sensitivity of the assay, we used reverse transcriptase PCR (RT-PCR) to detect the full-length coding-sequence (CDS) of PR-B using gene-specific primers in Huh7-S10-3 cells. Since the difference between PR-B and PR-A isoforms is that the PR-A lacks the first 164 amino acid residues of PR-B due to translation from a second start codon, only the full-length PR-B CDS (2,799 bp in size) was analyzed by RT-PCR. We used the full-length CDS (1,128 bp) of the G protein-coupled estrogen receptor (GPER) as a PCR control. We used primer-specific annealing temperature (PR-B Ta 53°C and GPER Ta 57°C) and also a higher annealing temperature (Ta 60°C) to minimize potential nonspecific amplification. There was no amplification of the full-length PR-B CDS by RT-PCR, while the GPER control gene RT-PCR was positive (Fig. 1B), thus confirming that Huh7-S10-3 liver cells express only nonclassical progesterone receptor PGRMC-1, and -2, but not the classical progesterone receptor PR-A and PR-B.

### Progesterone enhances HEV replication at a concentration seen during pregnancy and in a dose-dependent manner.

**FIG 2:**
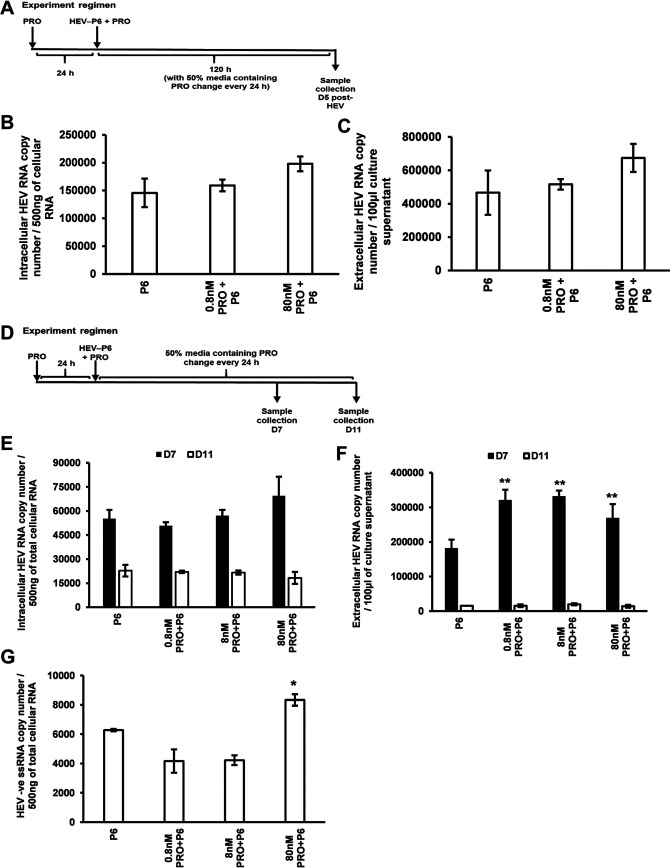
Progesterone enhances HEV replication at a concentration seen in the first trimester of pregnancy and in a dose-dependent manner. (A) Experimental regimen of progesterone treatment. (B) Intracellular HEV RNA levels. (C) Extracellular HEV RNA levels in HEV-P6 transfected Huh7-S10-3 cells, at day 5 (D5) post-HEV transfection, in the presence or absence of progesterone as determined by HEV RT-qPCR. (D) Experimental regimen of progesterone treatment during growth kinetics of HEV (D7 and D11). (E) Intracellular HEV RNA levels. (F) Extracellular HEV RNA levels, at D7 and D11 post-HEV transfection, in the presence of various concentrations of progesterone as determined by HEV RT-qPCR. Progesterone concentrations in final culture volume are 0.8 nM PRO = 0.25 ng/ml; 8 nM PRO = 2.5 ng/ml; 80 nM PRO = 25 ng/ml. (G) HEV negative-strand RNA levels in progesterone-treated cells compared to HEV-transfected cells without progesterone treatment as determined by HEV negative-strand RT-qPCR. Data represent average ± standard error of the mean (SEM) from panels B and C, representative of 3 independent experiments; for panels E, F, and G, *n* = 2 independent experiments; Student’s *t* test; *, *P* ≤ 0.05; **, *P* ≤ 0.01.

To determine the potential role of progesterone (PRO) in HEV replication, Huh7-S10-3 cells were pretreated with various concentrations of progesterone (0.8 nM PRO = 0.25 ng/ml, 8 nM PRO = 2.5 ng/ml, and 80 nM PRO = 25 ng/ml). At 24 h after progesterone pretreatment, the cells were transfected with the RNA transcripts of genotype 3 HEV-P6, and the transfected cells were cultured in the presence of progesterone (Fig. 2A). The HEV-P6 RNA-transfected Huh7-S10-3 cells without any progesterone treatment served as the control. We showed that pretreatment with 80 nM PRO resulted in increased levels of both intracellular and extracellular HEV-ORF2 RNA at 5 days posttransfection (D5) with HEV RNA transcripts (Fig. 2B and C).

Growth kinetics of HEV replication were also performed on day 7 (D7) and day 11 (D11) posttransfection (Fig. 2D). An increase in intracellular HEV RNA levels was observed in 80 nM PRO-treated liver cells compared to the control with no progesterone treatment (Fig. 2E), and a significant increase in the level of the extracellular HEV RNA was observed in the progesterone-treated cells on D7 (Fig. 2F). No significant difference was observed between the progesterone-treated cells and the control with no progesterone treatment at D11.

To further confirm that the increase in HEV RNA level at D7 we observed in these experiments was indeed due to an enhanced HEV replication and not due to the detection of any residual transfected HEV RNA, we tested the levels of the intracellular HEV negative-strand RNA in both progesterone-treated and untreated HEV-transfected Huh7-S10-3 cells at D5 post-HEV RNA transfection. Our results showed that the HEV negative-strand RNA levels were significantly increased in 80 nM PRO-treated cells compared to the control with no progesterone treatment (Fig. 2G). The 80 nM PRO concentration of progesterone represents a concentration typically seen during the first trimester of pregnancy (Table 1).

**FIG 3:**
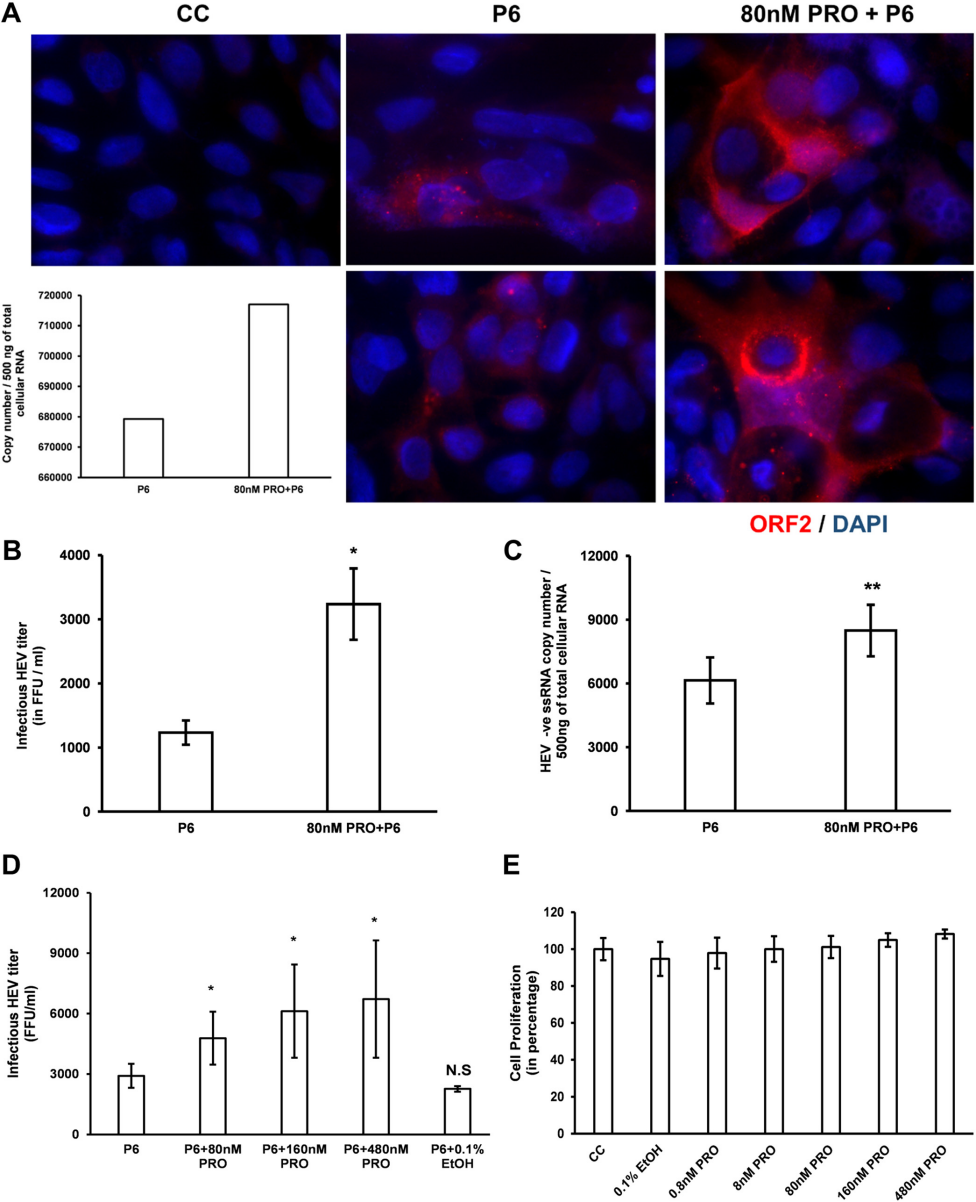
Progesterone pretreatment of Huh7-S10-3 human liver cell enhanced HEV replication as determined by IFA of HEV capsid protein and virus infectivity assay. (A) Representative images of immunofluorescence (two different fields) of HEV ORF2 capsid protein in HEV-P6 transfected Huh7-S10-3 cells, at D5 post-HEV transfection, in the presence or absence of 80 nM PRO. Nuclei are counterstained using DAPI. The inset bar diagram represents intracellular HEV RNA levels determined during the experiment. CC, “cell-control” without HEV transfection. (B and D) Infectious HEV titers as determined by an HEV infectivity assay. (C) HEV negative-strand RNA levels as determined by HEV negative-strand RNA RT-qPCR. (E) Cell proliferation level determined by a WST-1 assay. Data represent an average ± SEM from panels B (*n* = 2), C (*n* = 5), D (*n* = 3 independent experiments), and E (n = 6 replicates); Student’s *t* test; *, *P* ≤ 0.05; **, *P* ≤ 0.01 compared to P6. Progesterone final concentrations are 80 nM PRO = 25 ng/ml, 160 nM PRO = 50ng/ml, 480 nM = 150 ng/ml.

To more definitively demonstrate the effect of 80 nM PRO on HEV replication, we determined the infectious titer of HEV using the HEV infectivity assay, as well as the expression levels of HEV ORF2 capsid protein using immunofluorescence assay (IFA). Consistent with our PCR results, the IFA results showed an increased fluorescence in ORF2-positive cells in 80 nM PRO-treated HEV-transfected cells compared to the control without progesterone treatment (Fig. 3A). A significant increase in infectious HEV titers was also observed in the 80 nM PRO-treated cells (3,237.5 ± 556.6 focus-forming units [FFU]/ml; Fig. 3B) compared to the control without progesterone treatment (1,231.25 ± 189 FFU/ml). This observation was further supported by a significant increase in the HEV negative-strand RNA levels (Fig. 3C) in these samples as well. We also tested the HEV replication levels in cells pretreated with progesterone at concentrations typically seen during the second (160 nM PRO) and third (480 nM PRO) trimesters of pregnancy, respectively. Our results showed that pretreatment of Huh7-S10-3 cells with 160 nM and 480 nM concentrations of progesterone, respectively, lead to significant increases in infectious HEV titer compared to the control with no progesterone treatment (Fig. 3D). However, there was no statistically significant difference in HEV infectious titers among the 80 nM, 160 nM, and 480 nM concentrations of progesterone-treated samples. To rule out the potential effect of residual ethanol in the progesterone stock, we also used 0.1% ethanol (0.1% EtOH) treatment as a control during HEV culture. Our results showed that 0.1% EtOH treatment by itself did not affect HEV replication levels (Fig. 3D).

We also estimated cell proliferation under various progesterone treatment conditions using a water soluble tetrazolium salts-1 (WST-1) assay kit (Sigma, USA) as per the manufacturer’s protocol. Progesterone treatment, at various concentrations, did not increase Huh7-S10-3 cell proliferation (Fig. 3E). Taken together, these observations indicate that the increased infectious titer of HEV during 80 nM to 480 nM progesterone treatment is specific and that progesterone enhances HEV replication at a concentration seen during pregnancy and in a dose-dependent manner.

### Progesterone pretreatment is required for enhanced HEV replication in human liver cells.

We further examined whether progesterone-mediated enhancement of HEV replication is also time-dependent. Since HEV infectious titers among 80 nM, 160 nM, and 480 nM progesterone-treated cells did not significantly differ, we used the 80 nM progesterone concentration in all further experiments.

**FIG 4:**
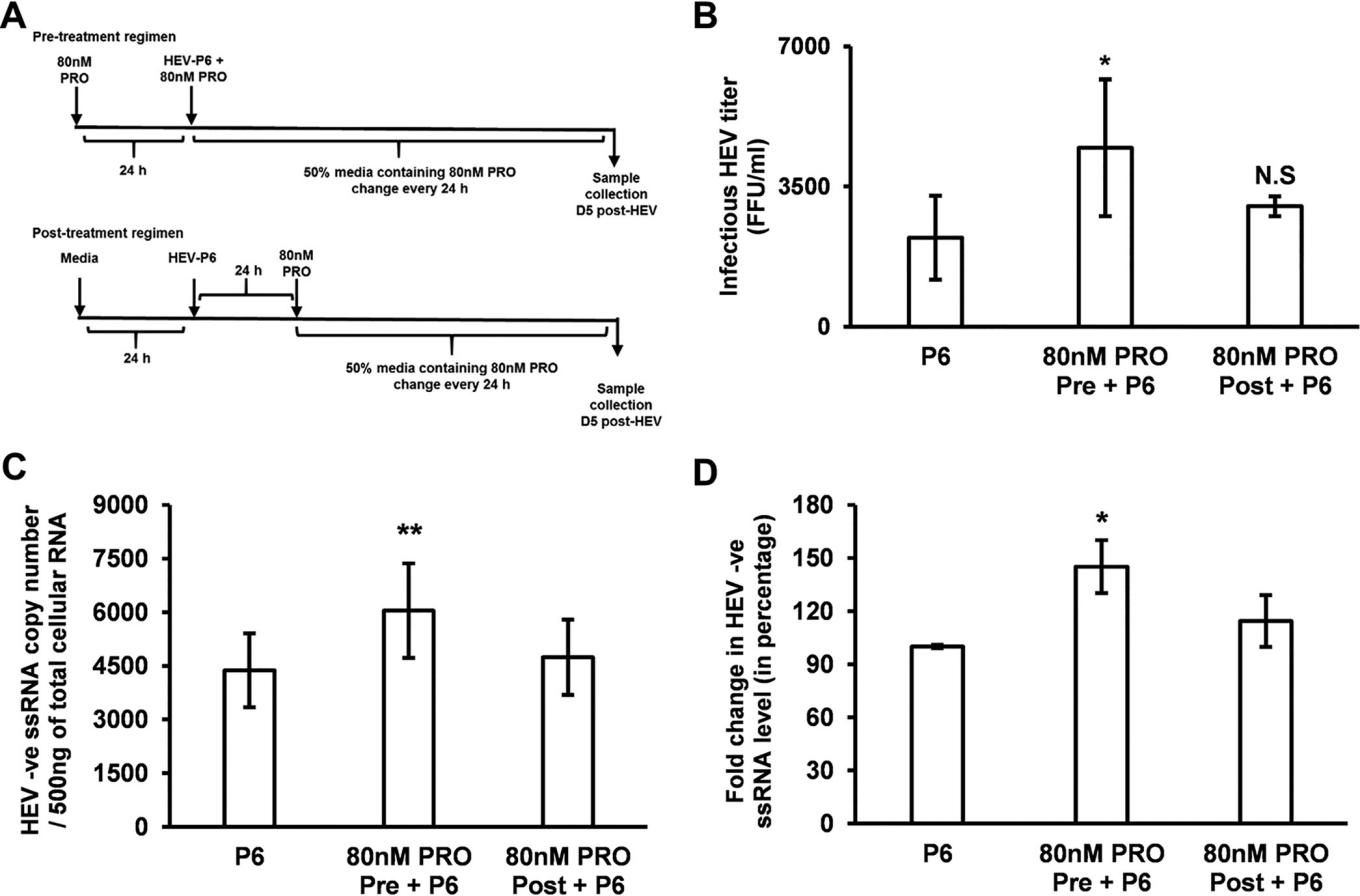
Progesterone pretreatment is required for enhanced HEV replication in human liver cells. (A) Experimental regimen of progesterone pretreatment (80 nM PRO Pre) and posttreatment (80 nM PRO Post). (B) HEV infectious titer. (C) HEV negative-strand RNA copy numbers. (D) Fold change (in percentage) in HEV-P6 transfected Huh7-S10-3 cells during pre- or posttreatment with progesterone. Data represent an average ± SEM from panels B (*n* = 3) and C and D (*n* = 7 independent experiments). Statistical analyses were performed with Student’s *t* test (panel B), one-way ANOVA with *post hoc* Tukey’s test (panel C), and the Friedman test (panel D) of differences among the multiple groups, which rendered a chi-square value of 7.71, which was significant (*P* = 0.02). *, *P* ≤ 0.05; **, *P* ≤ 0.01 compared to HEV-P6 by Student’s *t* test. 80 nM PRO = 25 ng/ml progesterone final concentration.

Huh7-S10-3 cells were treated with 80 nM PRO either pre- or post-HEV-P6 RNA transfection as described in Fig. 4A. The change in HEV infectious titer and the levels of HEV negative-strand RNA were measured using the HEV infectivity assay and reverse transcriptase quantitative PCR (RT-qPCR), respectively. As observed in a previous experiment, we found that the 80 nM PRO pretreatment led to a significant increase in the levels of HEV infectious titer and of negative-strand RNA compared to HEV RNA transfected cells without progesterone treatment. Importantly, the 80 nM PRO treatment did not affect the level of HEV replication when added at 24 h post-HEV RNA transfection (Fig. 4B to D), suggesting that progesterone pretreatment is essential for enhanced HEV replication. Further experimentation was undertaken to test if the proviral state established by progesterone is due to an inhibition of HEV-induced innate response.

### HEV infection induces IFN-λ1 response in human liver cells.

**FIG 5:**
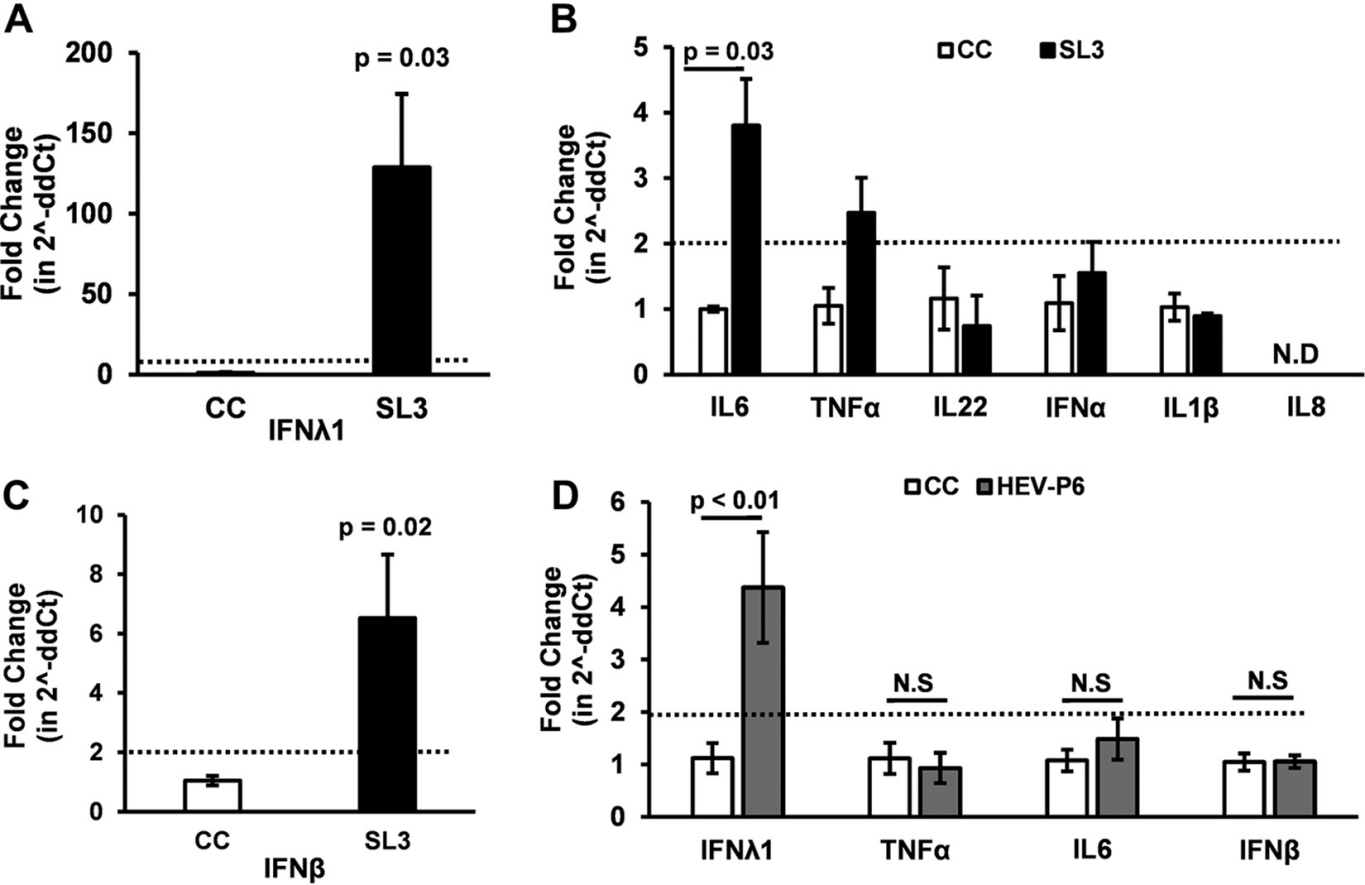
Innate cytokine mRNA profile induced by a known IFN inducer (HEV SL3, positive control) and by HEV-P6. (A) IFN-λ1 mRNA levels. (B) Other cytokine (IL-6, TNF-α, IL-22, IFN-α, IL-1β, and IL-8) mRNA levels. (C) IFN-β mRNA levels induced by HEV-P6 3′ UTR PAMP SL3-transfected (black-filled bars) Huh7-S10-3 cells compared to the mock cell control (CC, unfilled bars). (D) IFN-λ1, IL-6, TNF-α, and IFN-β levels in Huh7-S10-3 cells transfected with genomic RNA transcripts of HEV-P6 (gray-filled bars) compared to the mock cell control (CC). Cytokine mRNA levels were determined using gene-specific cytokine RT-qPCR. The fold change was calculated using the 2^-ddCt method. The data represent an average ± SEM from panels A and B (*n* = 2 independent experiments) and from panels C and D (*n* = 4 independent experiments); Student’s *t* test. N.D, not detected; N.S, nonsignificant.

We previously reported that the 3′ untranslated region (UTR) (HEV SL3, 169 nucleotides [nt]) of the genotype 3 HEV-P6 is a strong inducer of type III IFN response in Huh7-S10-3 liver cells (23); therefore, in this study we first determined the expression profile of various innate cytokines (type I IFN, type III IFN, tumor necrosis factor alpha [TNF-α], interleukin-22 [IL-22], IL-1β, IL-8, and IL-6), in HEV SL3-transfected Huh7-S10-3 liver cells. Any change, as determined by the Livak delta-delta Ct method (i.e., 2^-ddCt method), resulting in a 2- or more-fold increase was considered as an induction. The results showed that the HEV SL3 induced a 100-fold increase in type III IFN-λ1 mRNA levels (*P* = 0.03, Fig. 5A), a 3-fold increase in IL-6 mRNA levels (*P* = 0.03, Fig. 5B), and a 6-fold increase in IFN-β mRNA levels (*P* = 0.02, Fig. 5C) in Huh7-S10-3 cells. Although we also observed a 2-fold increase in the level of TNF-α mRNA (Fig. 5B), it was not statistically significant compared to the uninduced Huh7-S10-3 cell control (CC). No change was observed in the levels of other cytokine mRNAs, including IFN-α, IL-22, and IL-1β (Fig. 5B). The IL-8 mRNA level remained undetectable (Fig. 5B).

Based on the expression profile of the innate cytokines as demonstrated above in cells transfected with the known type III IFN inducer, HEV SL3, we then further quantified the amount of IFN-λ1, IFN-β, TNF-α, and IL-6 mRNA expression levels in Huh7-S10-3 liver cells transfected with the infectious RNA transcripts of the genotype 3 HEV-P6. We showed that, at 24 h posttransfection with capped HEV genomic RNA transcripts, there is a 4-fold increase in IFN-λ1 (*P* < 0.01) mRNA levels in Huh7-S10-3 cells, although no increase in IFN-β, TNF-α, and IL-6 mRNA levels was observed (Fig. 5D). Since the HEV-P6 strain induced a significant increase in IFN-λ1 levels in human liver cells, we subsequently tested whether progesterone modulates HEV-induced IFN-λ levels, thus possibly causing an enhanced HEV replication.

### Progesterone treatment had no significant effect on HEV-induced IFN-λ response.

**FIG 6:**
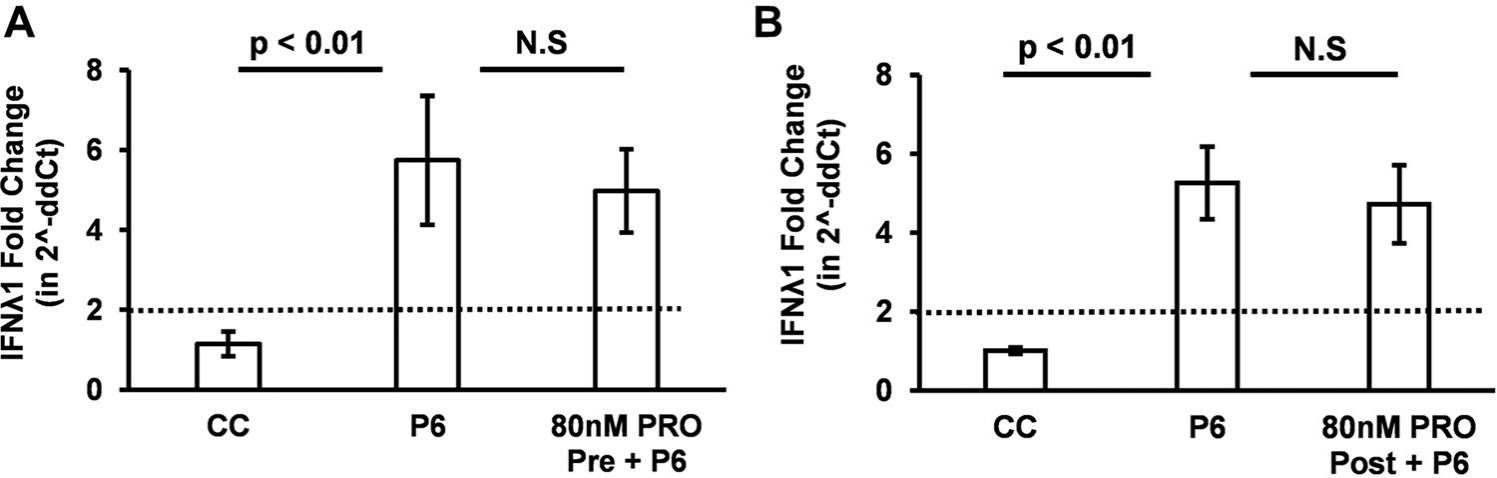
Progesterone treatment had no effect on the HEV-induced IFN-λ1 innate immune response. (A) Progesterone pretreatment (*n* = 4 independent experiments); fold change in IFN-λ1 mRNA levels in HEV-P6 transfected Huh7-S10-3 cells. (B) Progesterone posttreatment (*n* = 4 independent experiments); fold change in IFN-λ1 mRNA levels in HEV-P6 transfected Huh7-S10-3 cells. The data represent an average ± SEM; Student’s *t* test. N.S, nonsignificant; 80 nM PRO = 25 ng/ml progesterone final concentration.

To determine if the progesterone-mediated enhancement of HEV replication is due to modulations in HEV-induced innate immune response by progesterone, we measured the levels of IFN-λ1 mRNA in Huh7-S10-3 liver cells transfected with genomic RNA transcripts of HEV-P6 in the presence or absence of progesterone. The results showed that both 80 nM PRO pretreatment (Fig. 6A) and 80 nM PRO posttreatment (Fig. 6B) had no significant effect on the HEV-P6-induced IFN-λ1 mRNA levels in Huh7-S10-3 cells compared with those of HEV-P6 transfected cells without progesterone treatment. This indicates that the 80 nM PRO pretreatment-mediated enhancement of HEV replication is not mediated by modulating the host cell IFN-λ response.

Additionally, we also tested the effect of progesterone on HEV SL3-induced IFN-λ mRNA levels. We included both a 0.1% EtOH control and a tRNA control to confirm that progesterone does not influence IFN-λ mRNA levels in Huh7-S10-3 cells even in the presence of a strong IFN stimulant (Fig. S1). Our results showed that progesterone treatment did not affect the HEV SL3-induced IFN-λ mRNA levels. As expected, the IFN-λ mRNA levels in the tRNA control remained at basal levels, and the 0.1% EtOH control did not have any effect on SL3-induced IFN-λ mRNA levels in Huh7-S10-3 cells (Fig. S1). Therefore, these results collectively indicate that progesterone does not directly affect IFN-λ induction in Huh7-S10-3 cells by HEV genome and/or HEV-SL3.

10.1128/mBio.01434-21.1 FIG S1 Progesterone treatment had no effect on the HEV-SL3-induced IFN-λ1 response in Huh7-S10-3 cells. (A to C) Fold change in IFN-λ1 mRNA levels in HEV-SL3-stimulated Huh7-S10-3 cells (A) in the presence or absence of 0.1% ethanol (EtOH), (B) during progesterone pretreatment, or (C) under progesterone posttreatment conditions. The data represent an average ± SEM of 2 independent experiments; Student’s *t* test; N.S, nonsignificant; 80nM PRO = 25 ng/ml progesterone final concentration. Download FIG S1, TIF file, 0.8 MB. Copyright © 2021 Sooryanarain et al. 2021 Sooryanarain et al. https://creativecommons.org/licenses/by/4.0/ This content is distributed under the terms of the Creative Commons Attribution 4.0 International license.

### Loss of PGRMC1/2 during progesterone treatment leads to a decrease in HEV replication, and an increase in HEV-induced IFN-λ1 levels via the extracellular signal-regulated kinase (ERK) pathway.

**FIG 7:**
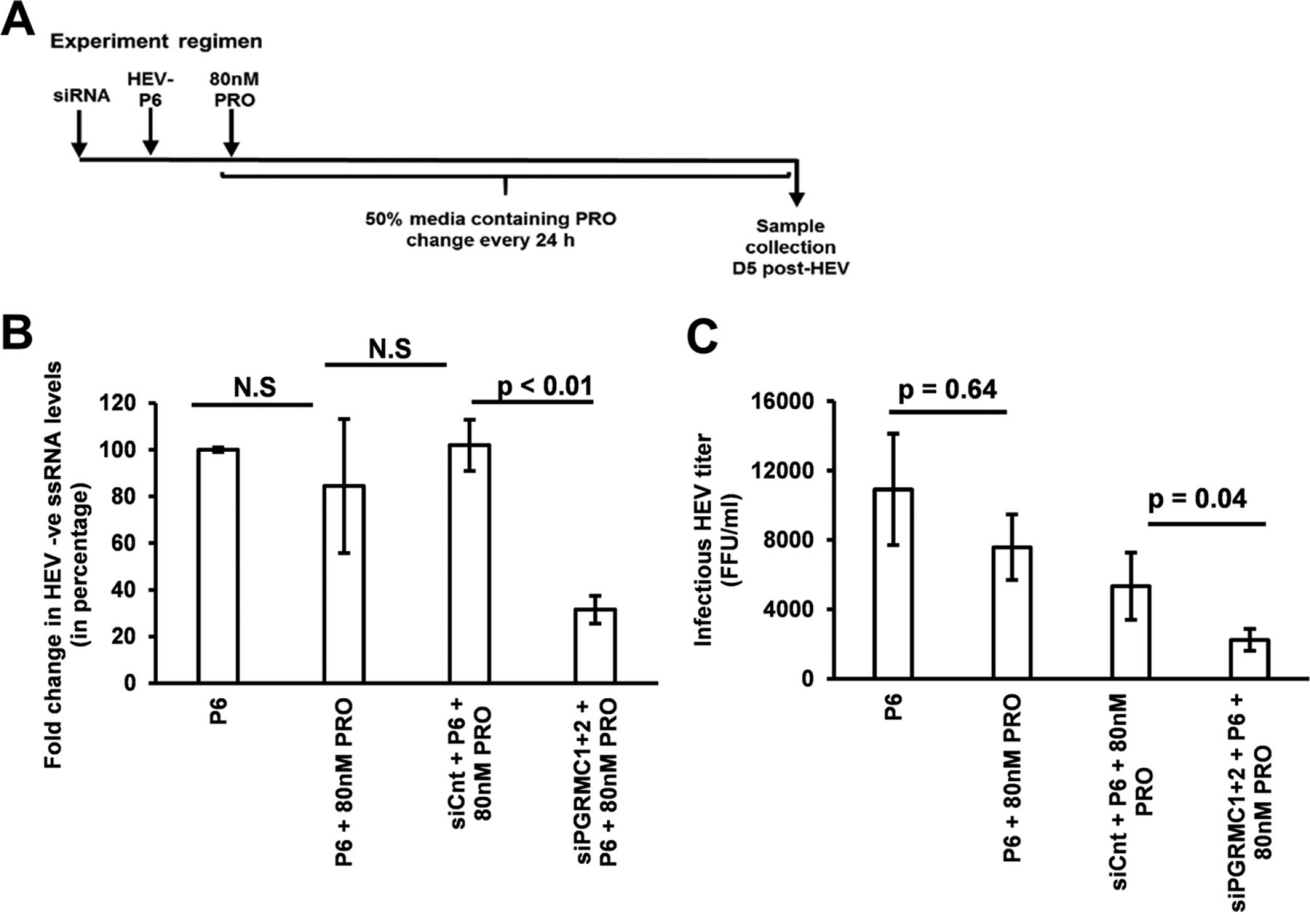
Loss of PGRMC1/2 by siRNA knockdown during progesterone treatment leads to a significantly decreased level of HEV replication. (A) Experimental regimen. (B) Percentage fold change in HEV negative-strand RNA levels; the Friedman test of differences among the multiple groups rendered a chi-square value of 8.40, which was significant (*P* = 0.03). (C) Infectious HEV titer (FFU/ml). The data represent an average ± SEM of panels B (*n* = 4) and C (*n* = 3 independent experiments); Student’s *t* test. N.S, nonsignificant; siCnt, scramble control siRNA transfected cells; siPGRMC1 + 2, PGRMC1/2 siRNA transfected cells; 80 nM PRO = 25 ng/ml progesterone final concentration.

**FIG 8:**
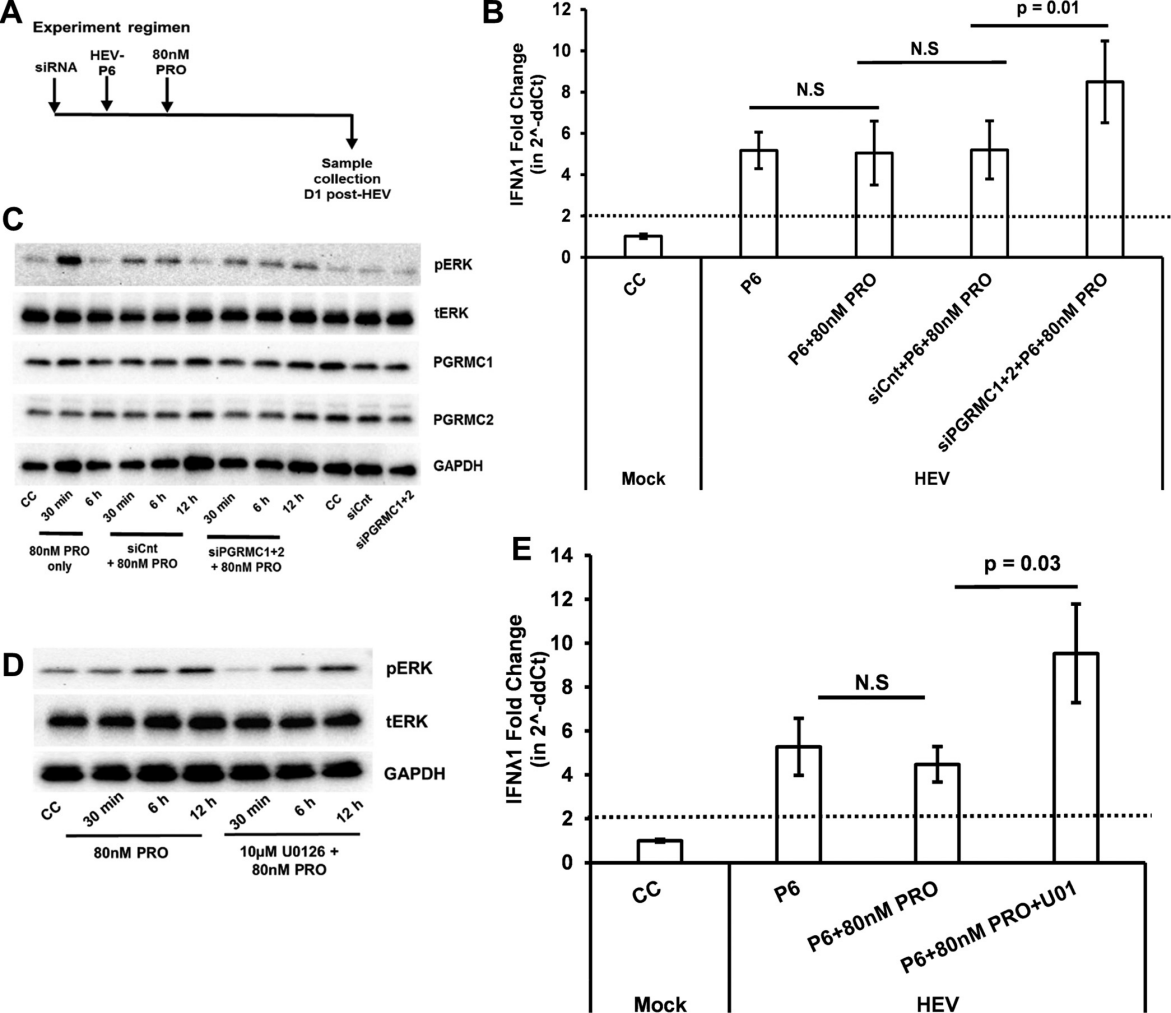
Loss of PGRMC1/2 by siRNA knockdown during progesterone treatment increased the levels of HEV-induced IFN-λ1 expression via ERK. (A) Experimental regimen. (B) Fold change in IFN-λ1 mRNA levels in various conditions tested. (C and D) Western blot analysis of phosphorylated ERK (pERK) and total ERK (tERK) levels during progesterone treatment. (C) PGRMC1/2 knockdown cells. (D) ERK inhibitor U0126 (U01) treated cells. (E) Fold change in IFN-λ1 mRNA levels in the presence or absence of ERK inhibitor U0126 (U01). The data represent an average ± SEM of 2 independent experiments; Student’s *t* test, N.S, nonsignificant; siCnt, scramble control siRNA transfected cells; siPGRMC1 + 2, PGRMC1/2 siRNA transfected cells; U01, 10 μM ERK inhibitor U0126; 80 nM PRO = 25 ng/ml progesterone final concentration.

Progesterone binds to nonclassical receptor PGRMC1/2 to mediate the nonclassical signaling pathway, and we have demonstrated that the Huh7-S10-3 liver cells express the nonclassical receptor PGRMC1/2 (Fig. 1). We showed that both progesterone pretreatment and posttreatment did not affect HEV-P6-induced IFN-λ mRNA levels (Fig. 6). However, only progesterone posttreatment did not affect HEV-P6 titers (Fig. 4). Therefore, we decided to further test whether PGRMC1/2 negatively regulates the HEV-induced IFN-λ response. PGRMC1/2 was knocked down via siRNA in Huh7-S10-3 cells, and the effect of PGRMC1/2 knockdown on HEV replication (Fig. 7A) and the HEV-induced IFN-λ response (Fig. 8A) were estimated during progesterone posttreatment. At 5 days post-HEV-RNA transfection, the HEV infectious titer and HEV negative-strand RNA levels were estimated to determine the HEV replication levels. As expected, progesterone posttreatment did not affect HEV replication; however, we found that knockdown of PGRMC1/2 using siRNA (siPGRMC1 + 2) during progesterone treatment led to a significant decrease in the level of HEV replication (*P* < 0.01) compared to control siRNA (siCnt)-treated samples with progesterone (Fig. 7B and C). We also measured the IFN-λ1 mRNA levels at an early time point under these conditions (Fig. 8A). The results showed that PGRMC1/2 knockdown in progesterone-treated cells leads to a significant increase in HEV-induced IFN-λ1 mRNA levels (*P* = 0.01) at 24 h post-HEV transfection compared to control siRNA-treated cells with progesterone (Fig. 8B).

The nonclassical pathway of progesterone signaling can induce various host kinases, including the extracellular signal-regulated kinase (ERK) pathway (15). Thus, in this study we determined the ERK pathway activation by progesterone under the PGRMC1/2 knockdown condition by estimating the ERK phosphorylation level using Western blot analysis. Our data showed that loss of PGRMC1/2 affected the kinetics of ERK phosphorylation (Fig. 8C). Progesterone treatment led to an acute increase in phosphorylated ERK at earlier time points (30 min, 6 h), while in the PGRMC1/2 knockdown cells, progesterone treatment led to low but sustained phosphorylated ERK levels. Therefore, we further measured the HEV-induced IFN-λ levels in the presence or absence of ERK inhibitor U0126 during the 80 nM progesterone treatment. We found that inhibition of the ERK pathway by 10 μM U0126 (Fig. 8D) led to a significant increase in HEV-induced IFN-λ mRNA levels under the progesterone posttreatment conditions (Fig. 8E). Therefore, the data suggest that loss of PGRMC1/2 receptors leads to an increase in HEV-induced IFN-λ1 expression levels at early time points via the ERK pathway and thus results in a reduced level of HEV replication.

## DISCUSSION

One of the unique features of HEV infection is the reported high mortality rate (up to 30%) and fulminant hepatic failures in HEV-infected pregnant women (6). However, the underlying mechanism of severe hepatitis during pregnancy remains elusive. An elevated level of progesterone is known to be produced in pregnant women, which is essential in maintaining pregnancy (13). Therefore, in this study, we aimed to determine whether the progesterone-mediated signaling pathway modulates HEV replication by influencing HEV-induced innate immune response in Huh7-S10-3 liver cells. We demonstrated that the Huh7-S10-3 liver cells express nonclassical progesterone receptor PGRMC1/2, but lack the classical progesterone receptors PR-A and PR-B, and that pretreatment with progesterone at 80 nM to 480 nM, which is the physiological concentration typically seen in the first- to third-trimester during pregnancy, significantly increased HEV replication in Huh7-S10-3 cells. We also showed that 80 nM progesterone did not modulate HEV-induced IFN-λ1 levels. Interestingly, loss of PGRMC1/2, the progesterone nonclassical receptor, resulted in a decreased level of HEV replication and an increased level of HEV-induced IFN-λ1 expression via the ERK pathway in Huh7-S10-3 liver cells.

Progesterone levels vary from 0.6 nM to 900 nM (i.e., <0.2 ng/ml to 290 ng/ml) depending on gender, menstrual cycle phase, and pregnancy (Table 1). In a clinical setting, increased HEV RNA levels and progesterone levels were reported in HEV-infected pregnant women (11, 12). It is noteworthy that thus far there is no experimental data to examine the role or mechanism of progesterone in HEV replication. It has been reported that medroxyprogesterone acetate (progesterone analog) treatment significantly increased HIV replication in human peripheral blood mononuclear cells (PBMCs) (24), although progesterone treatment did not appear to affect the hepatitis C virus (25, 26) replication in Huh7 liver cells. Since high mortality rates were seen in HEV-infected women especially during the third trimester, in this study we pretreated cells with 80 nM, 160 nM, and 480 nM PRO, which is the physiological progesterone range typically seen during the first, second, and third trimesters of pregnancy (Table 1), respectively, to assess its effect on HEV replication. We found that pretreatment of Huh7-S10-3 human liver cells with 80 nM to 480 nM PRO led to a significant increase in HEV infectious titers. Additionally, we did not observe any progesterone-induced cell proliferation under our experimental conditions. Therefore, the progesterone pretreatment-mediated increase in HEV replication is specific and is possibly due to a proviral state induced by progesterone pretreatment.

Steroid hormones can modulate cytokine responses to regulate virus replication indirectly and thus affect the disease severity. For example, ovariectomized mice treated with progesterone were highly susceptible to genital herpes simplex virus type 2 (HSV-2) infection and developed poor innate and adaptive immune responses as measured by lower levels of IFN-γ and IgG (27). Several other studies have also investigated the potential role of pregnancy or progesterone in different viral infections. The progesterone treatment did not offer protection against HSV-2 infection following systemic immunization (28). Pregnant mice experimentally infected with the influenza virus had an impaired overall antiviral immune response (29) and a dysregulated inflammatory response and upregulation of matrix metallopeptidase 9 (MMP9) (30). Exposure of HIV-infected PBMCs to a progesterone compound, medroxyprogesterone acetate, led to a significant increase in the expression of HIV coreceptor CCR5 in T cells in addition to the CD4/CD8 ratio (24). In monocyte-derived macrophage culture, a high concentration of progesterone, 64 nM, i.e., 20 ng/ml concentration, reduced HIV-induced proinflammatory responses (31). In HEV-infected pregnant women, impairment of Toll-like receptor (TLR)-mediated innate immune response is observed (22, 32–34). Previous studies have shown that HEV induces type III IFN (IFN-λ) production (20, 21). Interestingly, in this study, we did not detect any significant change in HEV-induced IFN-λ levels in Huh7-S10-3 liver cells in the presence or absence of progesterone treatment. It should be noted that most of the studies on progesterone-mediated immune modulation are based on the murine model (27, 29, 30) or human PBMCs in the HIV (24, 31) infection model. Therefore, it is possible that progesterone mediates indirect immunomodulatory effects through impairing activation of PBMCs in HEV infection, while enhancing viral RNA levels in hepatocytes in HEV-infected pregnant women.

We further explored the potential role and underlying mechanism of progesterone nonclassical receptor, PGRMC1/2, in HEV replication. We showed that the loss of PGRMC1/2 by siRNA knockdown during progesterone treatment led to a significant reduction in HEV replication. It is plausible that this may be due to the increased HEV-induced IFN levels via the ERK pathway in Huh7-S10-3 liver cells. Progesterone mediates nonclassical signaling through PGRMC1/2 (15), but the precise signal transduction mechanism by which progesterone mediates PGRMC1/2 signaling (35), or the role of PGRMC1/2 in IFN induction pathways, is not yet elucidated. Therefore, future in-depth mechanistic studies are warranted to determine the exact role of PGRMC1/2 and progesterone signaling in the HEV-induced IFN pathway. Interestingly, SH3 motifs and tyrosine kinase binding motifs have been predicted in PGRMC1/2 (35). It is known that HEV ORF3 can bind to SH3 domains to activate cellular kinases (36, 37), an aspect that merits future investigations as well.

In conclusion, in this study, we demonstrated that pretreatment of progesterone at a level seen during pregnancy significantly increased HEV replication in hepatocytes. Our data suggest that this may be through activation of the nonclassical PGRMC1/2 signaling pathway, rather than immunomodulation of HEV-induced interferon response. The results shed light on the potential underlying mechanism of severe hepatitis that has been reported in HEV-infected pregnant women and offered a potential new research direction in understanding the mechanism of HEV pathogenesis during pregnancy.

Due to the lack of an efficient cell culture system to study the genotype 1 HEV replication, we were unable to determine the effect of progesterone on genotype 1 HEV replication in this study, which is a limitation of the study. Another limitation of this study is the use of an *in vitro* system with a cell culture-adapted genotype 3 HEV to study the effect of progesterone on HEV replication. HEV-associated acute fulminant liver failure in pregnant women is predominantly caused by genotype 1 HEV infection (6). Sporadic cases of genotype 3 infection have been reported in pregnant women from Europe (38, 39). However, unlike genotype 1 HEV infection, genotype 3 HEV infection in pregnant women appears to resolve spontaneously without severe complication to the mother or fetus (6), even though experimental genotype 3 HEV infection in pregnant rabbits reportedly causes severe disease (7–9). A recent *ex vivo* study has shown that genotype 1 HEV replicated efficiently and significantly impaired type III IFN production in the decidual and placental explants, while genotype 3 HEV replicated poorly and did not impair type III IFN production (40). Additionally, the genotype 3 HEV-P6 strain used in this study has an insertion of human ribosomal protein sequence S17 in its genome, which enables it to adapt in cell culture (41), and whether the S17 sequence has any influence on the results from this study is unknown. Furthermore, an *in vitro* model to replicate an environment during pregnancy in humans is rather difficult, since various factors, including constant flux in bioavailability of hormones and interaction of progesterone with other hormones, influence the maintenance and/or progress of pregnancy term (13). It should also be noted that experimental infection of genotype 1 HEV in pregnant rhesus monkey (42) and genotype 3 HEV in pregnant sows (43) did not result in severe hepatitis as reported in pregnant women. Therefore, development of more robust *in vitro* and *in vivo* models that can mimic the severe manifestation of hepatitis E diseases during pregnancy would be essential to more definitively define the underlying mechanism of HEV-induced acute hepatic failure during pregnancy. Future studies are warranted to evaluate if progesterone affects disease pathogenesis of various other HEV genotypes using relevant animal models.

## MATERIALS AND METHODS

### Cells, HEV infectious clone, antibodies, siRNAs, and progesterone.

The Huh7-S10-3 cells are a subclone of Huh7 human hepatocarcinoma cells (44), which were kindly provided by Suzanne U. Emerson (NIH, Bethesda, MD). HepG2-C3A, and Huh7-S10-3 cells were maintained in Dulbecco’s minimal essential medium (DMEM; Gibco-Thermo Fisher, Massachusetts, USA) with 5% fetal bovine serum (FBS; Atlanta Biologicals-RnD Systems, Minnesota, USA), 1× antibacterial-antimycotic (Gibco-Thermo Fisher), and 1× minimal essential amino acids (Gibco-Thermo Fisher). The genotype 3 HEV (Kernow P6 strain) infectious cDNA clone used in this study was described previously (41). The HEV-P6 infectious genomic RNA was transcribed using an mMESSAGE mMACHINE T7 *in vitro* transcription kit (Invitrogen-Thermo Fisher). The rabbit anti-human PR-A, PR-B, PGRMC1, phospho-ERK (pERK), and ERK antibodies were purchased from Cell Signaling Technology (Danvers, MA, USA), and mouse anti-human PGRMC2 antibodies was procured from Santa Cruz Biotechnology (Dallas, TX, USA). The mouse anti-human GAPDH antibody was procured from Invitrogen-Thermo Fisher (Waltham, MA, USA). The scramble siRNA was purchased from Qiagen Sciences, Inc. (Germantown, MD, USA), and siRNAs (siPGRMC1 and siPGRMC2) were purchased from Santa Cruz Biotechnology. Cell-culture-grade progesterone was procured from Sigma (St. Louis, MO, USA); a stock of 800 μM concentration was made in 100% ethanol, and aliquots were stored at −80°C until use. The ERK inhibitor, U0126, was procured from Sigma; a stock of 1 mM concentration was made in 100% dimethyl sulfoxide (DMSO), and aliquots were stored at –80°C until use.

### Progesterone treatment of Huh7-S10-3 cells.

Since the physiological progesterone concentration in human serum varied from 0.6 nM to 900 nM, we used the following concentrations as representations: 0.8 nM for basal physiological concentration, 8 nM for ovulation and the luteal phase of the menstrual cycle, and 80 nM for the first trimester of pregnancy (Table 1). To avoid cross-interference of other steroids, care was taken to use phenol red-free media and charcoal-stripped FBS to culture Huh7-S10-3 cells. Accordingly, the Huh7-S10-3 cells were grown in phenol-red free DMEM with 25 mM HEPES, 5% charcoal stripped FBS, 1× antibacterial-antimycotic, and 1× minimal essential amino acids (Gibco-Thermo Fisher, Massachusetts, USA) for 48 h. The cells were then plated in a 12-well plate at a concentration of 0.15 × 10^6^ cells per well in a 1.2-ml volume of phenol-red free DMEM with supplements as mentioned above. Cell-culture-grade progesterone stock was serially diluted to a working stock 800 nM, 80 nM, and 8 nM in endotoxin-free, phenol-red free DMEM with supplements, and 130 μl of diluted progesterone working stock was added to each well to achieve a final progesterone concentration of 80 nM, 8 nM, and 0.8 nM, respectively, in the cell culture medium. The progesterone treatment regime used in each experiment is described in the figure legends. Progesterone was replenished every 24 h with a 50% medium change. The final ethanol concentration in cell culture never exceeded 0.1%.

### Western blot analysis.

Huh7-S10-3 cells treated with various concentrations of progesterone were collected at different time points using 1× RIPA buffer containing protease-phosphatase inhibitors. The cell lysate was clarified by centrifugation at 14,000 × *g* for 5 min at 4°C. Approximately 40 μg of clarified lysate was loaded per well and resolved using 4 to 20% SDS-PAGE gel. The separated proteins were then transferred onto a 0.45-μM polyvinylidene difluoride (PVDF) membrane and blocked using 5% bovine serum albumin (BSA) in PBST (0.1% Tween20 in phosphate-buffered saline [PBS]). The membrane was then probed using anti-PR-A (1:1,000 dilution), anti-PR-B (1:1,000 dilution), anti-PGRMC1 (1:1,000 dilution), anti-pERK (1:1,000 dilution), anti-ERK (1:1,000 dilution), anti-GAPDH (1:5,000 dilution) antibodies. Bovine anti-rabbit-horseradish peroxidase (HRP) (1:7,000 dilution) and goat anti-mouse-HRP (1:3,000 dilution) were used as the secondary antibody. The membrane was then developed using Luminol reagent (SCBT, California, USA) and imaged using a Bio-Rad imaging system (Bio-Rad, California, USA).

### RT-qPCR for quantification of cytokines in Huh7-S10-3 human liver cells.

Total cellular RNAs were extracted from Huh7-S10-3 cells using TRI Reagent (MRC, Ohio, USA) as per the manufacturer’s protocol. The extracted RNAs were then treated with Turbo-DNase (Invitrogen-Thermo Fisher, Massachusetts, USA) and precipitated using lithium chloride (Invitrogen-Thermo Fisher) to obtain the purified RNA. The cDNA was synthesized from the purified RNA (500 ng/20 μl reaction volume) using random hexamer and an ABI high-capacity cDNA kit from ABI-Thermo Fisher (Waltham, MA, USA). The mRNA levels of cytokines were quantified from the cDNAs using ABI Power Sybr green qPCR mix (ABI-Thermo Fisher) and cytokine gene-specific primers (Table 2). The qPCR conditions were 95°C for 2 min, followed by 40 cycles of 95°C for 5 s, 60°C for 10 s, and 72°C for 20 s.

**TABLE 2:** Oligonucleotide primers used in this study

ID^*a*^	Sequence (5′–3′)	Purpose
IFN-α FP	GCCATCTCTGTCCTCCATGAG	qPCR
IFN-α RP	TCACACAGGCTTCCAAGTCATT	qPCR
IFN-β FP	AGTAGGCGACACTGTTCGTG	qPCR
IFN-β RP	GCCTCCCATTCAATTGCCAC	qPCR
IFN-λ1 FP	AAAAAGGAGTCCGCTGGCTG	qPCR
IFN-λ1 RP	TCAGACACAGGTTCCCATCG	qPCR
TNF-α FP	GCTGCACTTTGGAGTGATCG	qPCR
TNF-α RP	GAGGGTTTGCTACAACATGGG	qPCR
IL-6 FP	CAATGAGGAGACTTGCCTGG	qPCR
IL-6 RP	TGGGTCAGGGGTGGTTATTG	qPCR
IL-8 FP	GGCTGGAGAGCTACACAAGA	qPCR
IL-8 RP	ACCCATCTCTCCTTGGGGTC	qPCR
IL-1β FP	AACCTCTTCGAGGCACAAGG	qPCR
IL-1β RP	GGCGAGCTCAGGTACTTCTG	qPCR
IL-22 FP	GCCCTATATCACCAACCGCA	qPCR
IL-22 RP	CGCTCACTCATACTGACTCCG	qPCR
RPS18 FP	TGATCCCTGAAAAGTTCCAGCA	qPCR
RPS18 RP	CTTCGGCCCACACCCTTAAT	qPCR
PR-B FP	ATGACTGAGCTGAAGGCAAAGG	CDS PCR
PR-B RP	CTTTTTATGAAAGAGAAGGGGTTTCACC	CDS PCR
GPER FP	ATGGATGTGACTTCCCAAGCCC	CDS PCR
GPER RP	CACGGCACTGCTGAACCTCACAT	CDS PCR
Tag+HEV-FP	CGGTCATGGTGGCGAATAAGGTGGTTTCTGGGGTGAC	HEV-ve strand cDNA synthesis
Tag	CGGTCATGGTGGCGAATAA	HEV-ve strand qPCR FP
HEV-FP	GGTGGTTTCTGGGGTGAC	HEV qPCR
HEV-RP	AGGGGTTGGTTGGATGAA	HEV qPCR
HEV-Probe	5′FAM/TGATTCTCAGCCCTTCGC/3′BHQ	HEV qPCR

a FP, forward primer; RP, reverse primer.

### RT-qPCR for quantification of the HEV RNA genome.

Total cellular RNAs (for intracellular viral RNA) and RNAs from cell culture supernatant (for extracellular viral RNA) were extracted using TRI Reagent as mentioned above. The levels of HEV RNA in the samples were quantified by using an established one-step HEV RT-qPCR protocol as described previously (45).

### RT-qPCR for quantification of HEV negative-strand RNA.

To detect the amount of intracellular HEV negative-strand RNA, cDNA was synthesized from the purified total cellular RNAs using Tag+HEV-FP primer (Table 2) and an ABI high-capacity cDNA kit. The HEV negative-strand qPCR was then carried out using the cDNA using a Sensifast No-ROX probe kit (Bioline-Thomas Scientific, New Jersey, USA) with primer pairs Tag and HEV-RP, and an HEV probe (Table 2). The qPCR conditions include 95°C for 2 min, followed by 40 cycles of 95°C for 5 s, and 60°C for 30 s. No RT control and no template controls were included during each qPCR run.

### Immunofluorescence assay (IFA).

Huh7-S10-3 liver cells transfected with RNA transcripts of the genotype 3 HEV-P6 infectious clone were fixed at 5 days posttransfection using 80% acetone and blocked using 10% goat serum in PBST. The cells were then stained using rabbit anti-HEV ORF2 antibody (1:500 dilution) (46). The donkey anti-rabbit-Rhodamine Red Fab (1:2,000 dilution; The Jackson Laboratory, Maine, USA) was used as the secondary antibody. The nuclei were counterstained using DAPI.

### HEV infectivity assay.

The titer of infectious HEV virions in a given sample was determined by the median tissue culture infectious dose (TCID) assay. Huh7-S10-3 cells were plated onto a 96-well plate. The test samples were serially diluted from a 1:5 dilution to a 1:50 dilution and seeded onto the cell monolayer. The samples were tested in duplicate. The cells were incubated with each diluted serum sample for 5 days at 34.5°C with 5% CO_2_. The cells were then fixed using 80% acetone, blocked with 10% goat serum in PBST, and stained with a chimpanzee anti-HEV antiserum (1:1,000 dilution), a kind gift from Suzanne U. Emerson, NIAID, NIH (47). Goat anti-human Alexa Fluor 488 (1:300 dilution) was used as the secondary antibody. DAPI was used to counterstain cell nuclei. Fluorescent foci were counted in cells for each dilution, and the focus-forming units (FFU)/ml was calculated as the infectious titer.

### Statistical analysis.

Statistical comparison was performed using JMP Pro 15 (Cary, NC, USA). Analysis of variance (ANOVA) with a *post hoc* Tukey test was used. The Friedman rank test was used for values represented in percentage. Paired, two-tailed, Student’s *t* test was used to compare a specific group with its corresponding control. A value of *P* ≤ 0.05 was considered significant.
