# Supplementary figures and images for: Progesterone-Mediated Enhancement of Hepatitis E Virus Replication in Human Liver Cells
Source: mBio. 2021 Jun 22;12(3):e01434-21. doi: 10.1128/mBio.01434-21 (PMC8262892; doi:10.1128/mBio.01434-21)

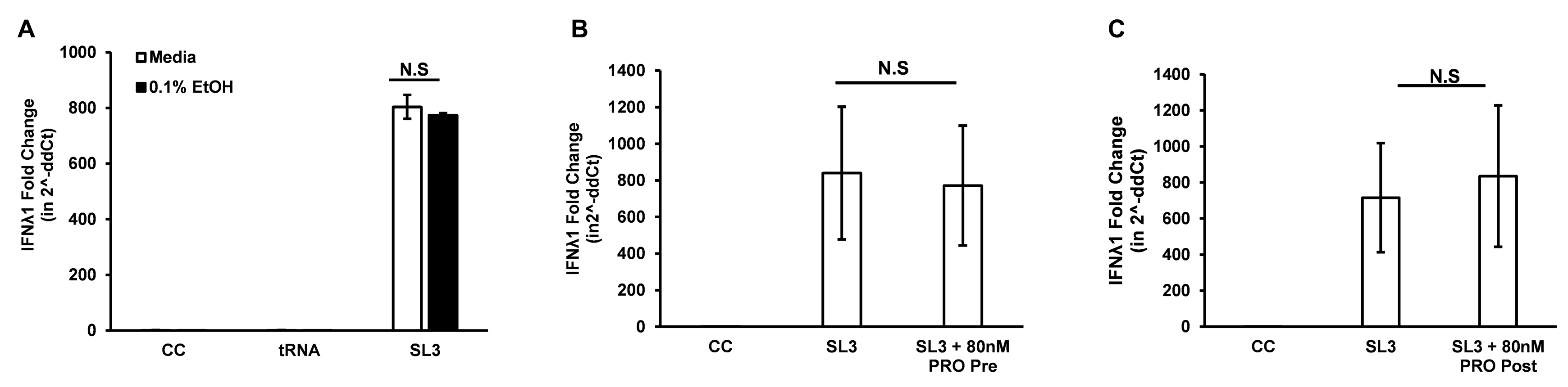

Supplement: FIG S1 [file mbio.01434-21-sf001.tif]
